# Impact of Ketamine on Quality of Recovery after Laparoscopic Surgery: A Single-Centre Single-Blinded Trial Using the QoR-15 Questionnaire

**DOI:** 10.1155/2023/8890025

**Published:** 2023-01-20

**Authors:** Helder Pereira, Maria Inês Graça, Diana Fonseca, Alfredo Mendes-Castro, Fernando Abelha

**Affiliations:** ^1^Department of Surgery and Physiology-Faculty of Medicine, University of Porto, Alameda Professor Hernâni Monteiro, Porto, Portugal; ^2^Anaesthesia Department, Centro Hospitalar Universitário de São João, Alameda Professor Hernâni Monteiro, Porto, Portugal; ^3^Department of Community Medicine, Information and Health Decision Sciences-Faculty of Medicine, University of Porto, Alameda Professor Hernâni Monteiro, Porto, Portugal

## Abstract

**Background:**

The quality of recovery is a cluster of patient-related outcomes that emphasise not only pain but different physical and emotional dimensions. Traditionally, ketamine is used to improve postoperative analgesia and avoid opioid consumption and opioid-related side effects.

**Objective:**

The present study sought to evaluate if intraoperative ketamine administration (as a part of multimodal analgesia) influences the quality of recovery after laparoscopic surgery.

**Design:**

A prospective two-armed, single-blinded trial. *Settings*. Tertiary single-centre trial between July 2021 and January 2022. *Patients*. From the 146 patients initially admitted to the study, 127 patients were enrolled, 60 in the ketamine group (group K) and 67 in the control group (group NK). *Intervention*. Both groups received a rigid intraoperative anaesthesia protocol; furthermore, in group K, 0.5 mg/kg of the ideal body weight of ketamine was administered. *Main Outcome Measures*. The primary outcome was to evaluate the effect of ketamine administration on the postoperative quality of recovery using the Portuguese version of the Quality of Recovery-15 (QoR-15) Questionnaire 24 h after surgery. The total score and minimal clinically significant difference (MCID) of the QoR-15 were compared. Other variables were also assessed such as the presence of emergence delirium (ED), the Numeric Rating Scale (NRS) for pain, and the presence of postoperative nausea and vomiting (PONV).

**Results:**

A total of 127 patients were allocated to the study groups, 60 in group K and 67 in group NK. Regarding the primary outcome, no differences were found in individual categories (15 items) and in the total score of QoR-15 (*p*=0.214). Concerning improvement (MCID ≥ 8) or worsening (MCID ≤ 8) in quality of recovery, no difference was found between the groups (24 vs. 32 and 6 vs. 6; *p*=0.776). Finally, no difference was found in secondary postoperative outcomes including ED (*p*=0.55), NRS (*p*=0.401), and PONV (*p*=0.55).

**Conclusion:**

In this study, the administration of ketamine in laparoscopic surgery had no impact on the quality of recovery 24 h after surgery. This trial is registered with NCT03724019.

## 1. Introduction

Recovery after surgery and anaesthesia is complex and depends on several factors based on patient, surgical, and anaesthetic characteristics, as well as the presence of numerous adverse sequelae or medication side effects [1, 2]. Recent studies evaluating recovery after anaesthesia and surgery have focused primarily on physiological endpoints, recovery times, and the incidence of adverse events, such as major morbidity and mortality. Although these parameters are essential and should be measured, they mostly ignore the patient's perspective [3]. This perspective, or patient-related outcomes, can be assessed with tools designed for that purpose, such as the Quality of Recovery-15 (QoR-15) Questionnaire. The QoR-15 provides an easy-to-use method to measure the quality of a patient's recovery from surgery and anaesthesia. It is an 11-point numerical rating scale that leads to a minimum score of 0 (very poor recovery) and a maximum score of 150 (excellent recovery) and has 15 questions (15 items) to evaluate five dimensions: pain, physical comfort, physical independence, psychological support, and emotional state [2, 4]. For QoR-15, it was established that the minimal clinically important difference (i.e., the minimal change in the score that would indicate a meaningful change in the health status) is an 8-point variation in the total score [5]. Using the patient's related outcomes scales, any intervention (therapeutical, surgical, etc.) can be assessed rigorously, objectively, (clinical's perspective), and subjectively (patient's perspective).

Ketamine is a potent inhibitor of NMDA (N-methyl-D-aspartate) receptors and an agonist of *µ* opioid receptors (responsible for ketamine's analgesic proprieties) [6]. It is used as an induction drug and as a component of a multimodal analgesia protocol to decrease postoperative opioid consumption [7]. Recently, ketamine was approved as an antidepressant [8]. When used as an adjunct in multimodal analgesia protocols, the recent literature suggests that a low dose of ketamine (<1 mg/kg) is associated with fewer side effects and is effective in decreasing postoperative opioid consumption and opioid-related side effects (nausea and vomiting) [7, 9, 10]. However, even when ketamine is used in low doses, ketamine's side effects (mainly visual and auditory hallucinations, depersonalisation, and changes in body perception) may influence the patient's perception of their quality of recovery [6]. Few studies have assessed the impact of intraoperative administration of ketamine on the quality of recovery, and the majority have used the 40-item Quality of Recovery (QoR-40) Questionnaire [7, 11, 12]. Therefore, this study aimed to evaluate if ketamine administration during anaesthesia induction impacts the quality of recovery after laparoscopic surgery under fentanyl-halogenated-based anaesthesia using the Portuguese version of the QoR-15 Questionnaire.

## 2. Methods

### 2.1. Ethics

Ethical approval for this study (certification number: 306/21) was provided by the Ethical Committee of Centro Hospitalar e Universitário São João, Porto, Portugal (Chairperson Prof Dr. Filipe Almeida) on 17 September 2021.

### 2.2. Study Design

A prospective two-armed, single-centre single-blinded trial was performed at the Anaesthesiology Department of Centro Hospitalar e Universitário São João (Porto, Portugal) and was registered at https://www.clinicaltrials.gov (number. NCT03724019; date of registration October 2018; principal investigator H.P.). The study was conducted in accordance with the Declaration of Helsinki. All collected data were saved anonymously, and informed consent was requested from all patients. This trial was performed according to the CONSORT guidelines.

### 2.3. Sample

Between July 2021 and January 2022, patients aged between 18 and 65 years, with physical status from the American Society of Anaesthesiology (ASA) I, II, or III (only if due to morbid obesity), who were scheduled for laparoscopic surgery for sleeve gastrectomy, salpingectomy, or cholecystectomy and admitted to postanaesthesia care unit (PACU) at Centro Hospitalar e Universitário São João, were eligible. The patient selection was not consecutive as the selection occurred only on the days the senior investigator was present. The exclusion criteria were patient refusal, incapacity of providing informed consent, history of drug addiction or alcohol abuse, pregnancy, urgent surgery, psychiatric pathology, use of medications not contemplated in the anaesthetic protocol, conversion to laparotomy, existence of adverse anaesthetic events in the intraoperative or anaesthetic recovery, and patients with a Revised Cardiac Risk Index (RCRI) > 1 [13]. The senior investigator allocated patients into two groups: group K, ketamine 0.5 mg/kg of the ideal body weight at anaesthesia induction or group NK, the control group. The allocation by study groups was sequential, following the scheme K–NK–K–NK. Before the surgery, the senior investigator informed the anaesthesiologist in charge if the patient was enrolled to the study. At that time, the appropriate study syringe was provided to the anaesthesiologist in charge.

### 2.4. Obtaining Data and Definitions

All patients were interviewed by a research team member the day before surgery. A small interview was conducted to obtain consent, collect the medical history, and perform the QoR-15 survey (Supplementary Material S1). Clinical risk factors (history of ischemic heart disease, history of compensated or prior heart failure, history of cerebrovascular disease, diabetes mellitus, and renal insufficiency) and the surgical risk were evaluated according to the cardiac risk stratification for noncardiac surgical procedures of the 2007 guidelines on Perioperative Cardiovascular Evaluation and Care for Noncardiac Surgery of the American College of Cardiology/American Heart Association Task Force on Practice [14]. The patient's anthropometric measures were recorded, and body mass index (BMI) (kg/m^2^) was calculated. Physical status ASA, RCRI, usual medication, presence of hypertension, type 2 diabetes mellitus, obesity, dyslipidaemia, respiratory pathology, depression, hypothyroidism, and the use of benzodiazepines at home were also collected. As a primary outcome of this study, the quality of recovery was evaluated using the Portuguese version of the Quality of Recovery 15-item (QoR-15) Questionnaire before surgery (D0) and 24 h after surgery (D1) [4]. The QoR-15 Questionnaire evaluates five dimensions: pain (2 items), physical comfort (5 items), physical independence (2 items), psychological support (2 items), and emotional state (4 items) [2]. The QoR-15 total score ranges from 0 (poorest recovery) to 150 (best recovery). The minimal clinically important difference (MCID) was defined as a positive (improved recovery) or negative (worsened recovery) variation in 8 points between D0 and D1 [5].

For group K patients, a total of 0.5 mg/kg of ketamine (considering the patient's ideal body weight) was administrated after propofol administration. For both the groups, anaesthesia was provided following the study protocol as follows: administration of drugs adjusted to the patient's ideal body weight (propofol, fentanyl, rocuronium, and dexamethasone) and the use of inhalational anaesthetic sevoflurane or desflurane for maintenance. The anaesthesia depth was monitored with state entropy and a deep neuromuscular block guided by the post-tetanic count (PTC). For intraoperative analgesia, fentanyl 3–5 g/kg (up to 30 minutes to the end of surgery) was administrated. Normothermia was achieved (guided by oesophageal temperature), and maintenance fluids with 0.9% saline solution were administered according to the Holliday–Segar formula. Before the end of the surgery, paracetamol 1000 mg, ketorolac 30 mg, tramadol 2 mg/kg, morphine 0.5 mg/kg, and ondansetron 4 mg were administrated. Sugammadex was used to obtain reversed muscle relaxation (T4/T1 > 0.9 on the TOF monitor). Usually, patients' trachea was extubated in the operating room and transferred to the PACU. Upon arrival at the PACU, all subjects were given oxygen by nasal cannula or face mask.

At the PACU, the data collection sheet was completed for each patient with immediate postoperative vital signs, including pain evaluation (using the NRS, Numerical Rating Scale). In order to detect some postoperative side effects of ketamine administration, all patients were screened for emergence delirium (ED) using the Nursing delirium Screening Scale (Nu-DESC) (Supplementary Material S2) and considered positive for ED with a Nu-DESC ≥2 points [15]. The postoperative analgesia administered, the incidence of nausea and vomiting, and the need for antiemetic medication were recorded at the time of discharge from the PACU. The length of hospital stay was at least 48 h.

### 2.5. Statistical Analysis

The sample size was calculated using the open access website, https://riskcalc.org/samplesize. According to the previous literature, the standard deviation (SD) of the total QoR-15 score in the Portuguese population is 14 [4]. An 8-point difference in the QoR-15 score represents the MCID that defines if the quality of recovery was improved (a positive 8-point difference) or worsened (a negative 8-point difference) [3, 5]. A minimum of 55 patients in each group were obtained (*α* = 0.05 and *β* = 0.20). Considering possible losses during the study (dropouts), the final sample constituted 138 patients. A descriptive analysis of variables was performed to summarise data. Group comparisons were performed using two independent sample *t*-tests (continuous variables), the Mann–Whitney *U* test (continuous variable with the non-normal distribution), and the chi-square test or Fisher's exact (dichotomous and ranked data). Differences were considered statistically significant when *p* < 0.05. An intention-to-treat analysis was also performed. Statistical analysis was performed using Statistical Package for Social Sciences (SPSS) version 27.0.

## 3. Results

**Figure 1:**
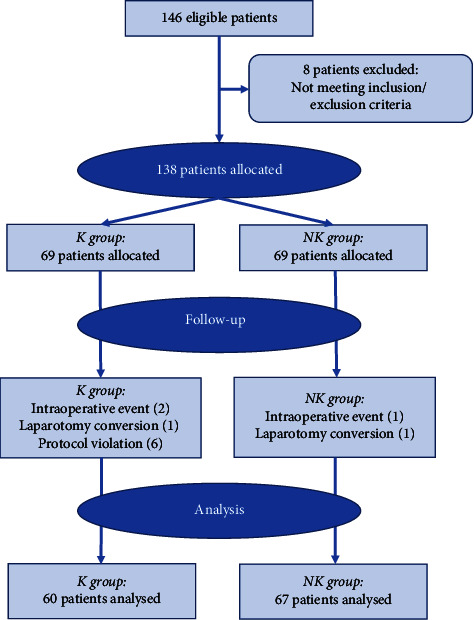
Flowchart of recruitable and included patients.

Of the 146 patients initially admitted to the study, 138 were enrolled and allocated to study groups, and the exclusion criteria excluded eight. During the study, eleven patients were excluded: nine from group K and two from the NK group due to intraoperative events (three cases of hemodynamic instability), surgical conversion to laparotomy (2 cases), or protocol violation (6 cases in the K group). Protocol violations occurred because ketamine administration did not occur during induction of anaesthesia. Finally, 60 patients from group K and 67 from group NK were analysed (Figure 1). Both the study groups were comparable regarding patient characteristics, namely, gender, age, type of laparoscopic surgery, BMI, physical status ASA, comorbidities, and benzodiazepines at home (Table 1).

Concerning the study's primary outcome, no difference was found between the groups for each category of QoR-15. Similar observation was found for the QoR-15 total score, with no significant differences found at the preoperative period (difference between the groups was one point (95% confidence interval (CI): −4.4–+3.2)), *p*=0.759 (Table 2) and 24 h after surgery (difference between the groups was 4 points (95% CI: −2.8–+12)), *p*=0.21 (Table 3).

Regarding the absolute variation in the QoR-15 score before and after surgery and QoR-15 MCID, no differences were found between the groups (Table 4).

About the other outcomes, ED incidence (*p*=0.55), pain after surgery (NIRS > 3, *p*=0.401), morphine requirement at PACU (*p*=0.137), PONV (*p*=0.55), and antiemetics requirement at PACU (*p*=0.166), no difference was found (Table 5).

An intention-to-treat analysis was performed (Tables 1–5) to confirm if all the results obtained would be different by including the protocol violation subjects in the analysis.

## 4. Discussion

This study showed that ketamine has no impact on the quality of recovery in some laparoscopic surgeries (sleeve gastrectomy, salpingectomy, and cholecystectomy): it neither improves nor worsens. A recovery with quality is not only a recovery without pain but also the patient's perception of emotional and psychological stability, physical comfort, and physical independence. Some QoR tools have been developed, such as QoR score, QoR-40, and QoR-15 [16, 17]. QoR-15 is a developed and validated short-form postoperative QoR score with fifteen questions that assess five domains of the patient-reported health status: pain, physical comfort, physical independence, psychological support, and emotional state [2]. The most used scale is the QoR-40, but QoR-15, newer and with comparable reliability statistics, may be more responsive to change [17]. Despite postoperative pain control being of significant importance, it is not the unique postoperative factor that plays a role in patient quality of recovery. Traditionally, opioids are seen as a playmaker for postoperative pain treatment. However, side effects, such as pruritus, nausea, and vomiting, may have a negative impact on the patient's perception of their recovery [12]. Ketamine has been studied as an adjunct to general anaesthesia to reduce postoperative pain. It has been established that low dose (<1 mg/kg) of ketamine improves postoperative pain. Moreover, the literature suggests a difference in the analgesic efficacy of ketamine depending on the administration time (with better results when ketamine is given during the anaesthesia induction) [6, 9]. In this study, regarding the QoR-15 patient's answers, the administration of ketamine during anaesthesia induction had no effect. No difference was found in all fifteen categories of the questionnaire and the total score. Also, the absolute QoR total score variation was not significant between the groups. Although all patients had a variation between preoperative and postoperative QoR results, only 50% of patients in group K and 57% in group NK had an absolute variation equal to or higher than 8 (MCID). In each group, six patients had an improved quality of recovery (the QoR-15 total score variation ≥ 8). The absolute number of patients in group NK with a worsened quality of recovery is higher than that of group K (32/67 > 24/60), but no difference was found regarding the QoR-15 MCDI (*p*=0.776) [5].

During the postoperative period at PACU, no difference in ED was found between the groups (*p*=0.55). ED is a common postoperative complication, with an incidence of 5–10% in the population, reaching 20% in some studies. Ketamine may contribute to emotion and mood regulation via inhibiting some neuronal pathways [7]. The assumption that the psychotomimetic effects of ketamine may lead to ED is still a debate, and despite the controversial literature, this link was not established [18–20].

A recent meta-analysis of twelve RCTs showed that subanesthetic intraoperative doses of ketamine have a beneficial effect on pain control in the immediate postoperative period (24 h), as they reduce the consumption of postoperative morphine and the intensity of pain following minor and major surgery. We did not find any difference in NRS and morphine requirement at PACU (*p*=0.401 and *p*=0.137) [21]. A theoretical hypothesis states that the presence of multimodal analgesia may partially suppress the MNDA receptor and block ketamine's central action [22]. A systematic review by Mcnicol et al. concluded that important factors might affect the efficacy of ketamine to improve postoperative analgesia, mainly inadequate dose or wrong time of administration [10]. Nonetheless, in our study, the analgesic protocol is largely multimodal (with paracetamol, ketorolac, tramadol, and morphine) and the scope for improving postoperative analgesia when adding ketamine is narrow.

Finally, PONV is a frequent complication that negatively influences the quality of recovery. Ketamine is a valid analgesic alternative to opioids to avoid opioid-related nausea and vomiting in the postoperative setting [6]. As for other proprieties of ketamine, its influence on PONV has contradictory results [23]. Recently, a systematic review concluded that ketamine reduced PONV [24]. In this study, the proportion of patients with PONV and antiemetics requirements at PACU was comparable between the two groups (*p*=0.55 and *p*=0.166).

We identify some limitations in our study. First, it would be preferable to conduct a randomized, double-blinded trial to avoid bias in enrollment and assessment of the subjects. This study was single-blinded: the senior investigator knew the assignment of the next subject prior to enrollment in the study, and this could have resulted in significant bias in terms of enrollment into the two groups. All the other participants—the study team who performed the QoR-15 questionnaire, anaesthesiologist in charge, and PACU nurses—were blinded to the treatment administered to the patients. Moreover, the anaesthesiologist in charge had a predefined anaesthesia protocol (confirmed with the anaesthetic chart), and the postoperative data recorded by the investigation team are clinically objective. The choice for a pre-established allocation scheme instead of randomization was due to some local ethical restrictions.

Second, the timing of the postoperative evaluation of QoR-15 (24 h after surgery). Maybe further evaluation would have been important at 48 hours or some days after surgery because the long-term effects of perioperative ketamine on patients are not well defined. Third, the patients recruited in this study were relatively healthy, and an upper age limit was set. The exclusion of patients with more comorbidities and more susceptible to poor recovery quality probably affects this study's external validity. Further studies should concentrate on elderly people with comorbidities to extend the universality. Additionally, we only analysed three types of laparoscopic surgery (sleeve gastrectomy, salpingectomy, and cholecystectomy): these laparoscopic procedures are relatively quick and similar. Results should not be extended to other intra-abdominal laparoscopic procedures. Finally, the study was designed to detect differences in the primary outcome and may not be sufficient to detect differences in the other variables analysed (the study may have been underpowered for some clinically significant effects). The adoption of a rigid anaesthetic protocol aimed to limit the presence of confounding factors.

This study is one of the first to enroll patients submitted to fentanyl-halogenated-based anaesthesia (most of the studies in the literature are remifentanil-intravenous based) to evaluate the postoperative effects of ketamine [12, 21]. Most studies use the variation of QoR-40 to evaluate the quality of recovery; we used the QoR-15 Questionnaire (comparable reliability statistics with QoR-40) and analysed the absolute score variation and the MCID to confirm the clinical significance of variations obtained [17].

In this study, the administration of ketamine in laparoscopic surgery had no impact (neither improves nor worsens) on the quality of recovery 24 h after surgery. There was no difference between the groups in each of the 15 categories of the QoR-15 and QoR-15 total scores. Regarding QoR-15 MCID, both the groups were comparable. This finding may highlight the importance of evaluating the postoperative results as a whole and not separately; as we saw in this study, some of the theoretical postoperative benefits effects of ketamine (found in an individual analysis) seem to be diluted when the evaluation is global.

## Figures and Tables

**Table 1:** Patient characteristics and preoperative data (*n* = 127).

Variable	NK Group (*n* = 67)	K Group (*n* = 60)	*p*-value
*Gender*			
Male	22 (32.8)	27 (45)	0.16^1^

Female	45 (67.2)	33 (55)

*Age*	47 ± 11.1	46.2 ± 9	0.708^2^

*Surgery*			
Cholecystectomy	20 (29.9)	20 (33.3)	0.764^1^
Sleeve gastrectomy	24 (35.8)	23 (38.3)	
Gynaecology	23 (34.3)	17 (28.3)	

*Body mass index (kg/m* ^*2*^)	29.3 ± 7.3	30 ± 6.5	0.531^2^

*Physical status ASA*			
I	16 (23.9)	15 (25)	0.799^1^
II	43 (64.2)	40 (66.7)	
III	8 (11.9)	5 (8.3)	

*Associated pathology*			
Obesity	31 (46.3)	31 (51.7)	0.543^1^
Hypertension	18 (26.9)	19 (31.7)	0.552^1^
Dyslipidaemia	13 (19.4)	16 (26.7)	0.33^1^
Type 2 diabetes mellitus	8 (11.9)	10 (16.7)	0.446^1^
Respiratory pathology	7 (10.4)	5 (8.3)	0.684^1^
Hypothyroidism	6 (9)	5 (8.3)	0.901^1^
Depression	6 (9)	4 (6.7)	0.748^3^

Benzodiazepines at home	13 (19.4)	15 (25)	0.448^1^

Data presented as mean ± standard deviation or *n* (%). Data were analysed using the ^1^chi-square test, ^2^*T*-test or ^3^fisher's exact test. ASA, American Society of Anaesthesiology.

**Table 2:** Preoperative Quality of Recovery-15 (QoR-15) scores.

QoR-15	NK group (*n* = 67)	Per protocol analysis (*n* = 127)		Intention-to-treat analysis (*n* = 133)	
		K group (*n* = 60)	*p* value^1^	K group (*n* = 66)	*p* value^1^
Item 1, breathing	9.8 (0.8)	9.7 (0.9)	0.175	9.7 (1)	0.148
Item 2, food	9 (2)	8.9 (2.3)	0.553	8.9 (2.2)	0.616
Item 3, rest	8.4 (2.4)	8.4 (2)	0.661	8.3 (2)	0.587
Item 4, sleep	7.1 (2.5)	7 (2.6)	0.92	7 (2.6)	0.936
Item 5, hygiene	9.9 (0.1)	9.9 (0.4)	0.257	9.9 (0.4)	0.301
Item 6, communication	9.9 (0.2)	9.9 (0.6)	0.64	9.9 (0.6)	0.582
Item 7, support	9.3 (2.3)	9.6 (1.2)	0.747	9.6 (1.2)	0.779
Item 8, return to work	9.4 (1.9)	9.4 (1.4)	0.911	9.4 (1.4)	0.936
Item 9, feeling in control	9.5 (1.1)	9.5 (1.2)	0.869	9.5 (1.2)	0.77
Item 10, well-being	8.9 (1.7)	8.7 (1.7)	0.381	8.8 (1.7)	0.435
Item 11, moderate pain	8.9 (1.8)	8.9 (2.4)	0.259	8.9 (2.3)	0.205
Item 12, severe pain	9.9 (0.7)	9.7 (1.1)	0.22	9.7 (1)	0.282
Item 13, nausea/vomiting	9.6 (1.2)	9.8 (1.8)	0.351	9.8 (1.7)	0.281
Item 14, anxiety	7.4 (2.1)	7 (2.4)	0.314	6.9 (2.3)	0.181
Item 15, depressed	8.9 (2)	9.1 (1.7)	0.782	9 (1.7)	0.839
Total score	136 (11)	135 (10)	0.759	135 (10)	0.42

Data are presented as the mean (SD). ^1^Mann–Whitney *U* test.

**Table 3:** Quality of Recovery-5 (QoR-15) scores 24 h after surgery.

QoR-15	Group NK (*n* = 67)	Per protocol analysis (*n* = 127)		Intention-to-treat analysis (*n* = 133)	
		Group K (*n* = 60)	*p* value^1^	Group K (*n* = 66)	*p* value^1^
Item 1, breathing	9.3 (1.8)	9.6 (1)	0.57	9.6 (1)	0.42
Item 2, food	6.4 (4.3)	6.8 (4.1)	0.604	6.8 (4)	0.597
Item 3, rest	7.9 (3.1)	8.5 (2.2)	0.591	8.5 (2.1)	0.519
Item 4, sleep	7.3 (3.5)	8.1 (2.7)	0.337	8.1 (2.8)	0.304
Item 5, hygiene	8 (3.7)	8.6 (3)	0.614	8.6 (3)	0.578
Item 6, communication	9.5 (1.5)	9.8 (0.7)	0.461	9.8 (0.7)	0.356
Item 7, support	9.4 (1.9)	9.5 (1.6)	0.674	9.4 (1.9)	0.628
Item 8, return to work	6.2 (3.8)	6.6 (3.5)	0.502	6.3 (3.5)	0.732
Item 9, feeling in control	8.2 (2.8)	8.6 (2.2)	0.987	8.5 (2.3)	0.851
Item 10, well-being	7.7 (2.6)	8.6 (1.9)	0.093	8.6 (1.9)	0.071
Item 11, moderate pain	7.1 (2.1)	6.6 (2)	0.171	6.6 (2)	0.195
Item 12, severe pain	9.4 (1.2)	9.4 (1.4)	0.837	9.4 (1.3)	0.872
Item 13, nausea/vomiting	9.3 (1.3)	9.1 (1.8)	0.951	9.1 (1.9)	0.791
Item 14, anxiety	8.3 (1.6)	8.6 (2.1)	0.311	8.5 (2.1)	0.394
Item 15, depressed	9 (1.9)	9.3 (1.6)	0.177	9.2 (1.6)	0.23
Total score	123 (23)	127 (17)	0.214	127 (18)	0.514

Data are presented as the mean (SD). ^1^Mann–Whitney *U* test.

**Table 4:** Postoperative data.

Variables	NK group (*n* = 67)	Per protocol analysis (*n* = 127)		Intention-to-treat analysis (*n* = 133)	
		K group (*n* = 60)	*p* value	K group (*n* = 66)	*p* value
QoR-15 |variation|	17.8 ± 18	12.5 ± 14	0.183^1^	12.6 ± 14.6	0.603^1^
QoR-15 MCID:					
≤8 points	32 (47.7)	24 (40)	0.776^2^	26 (39.5)	0.904^2^
No MCID	29 (43.3)	30 (50)		34 (51.5)	
≥8 points	6 (9)	6 (10)		6 (9)	

Data presented as the mean + standard deviation or *n* (%). ^1^Mann–Whitney *U* test. ^2^Chi-square test. MCID, minimal clinically important difference.

**Table 5:** Postoperative data.

Variables	NK group (*n* = 67)	Per protocol analysis (*n* = 127)		Intention-to-treat analysis (*n* = 133)	
		K group (*n* = 60)	*p* value^1^	K group (*n* = 66)	*p* value^1^
Emergency *Delirium*	9 (13.4)	6 (10)	0.550	7 (11)	0.616
Pain after surgery:					
NRS ≤ 3	41 (61.2)	41 (68.3)	0.401	45 (68.2)	0.399
NRS > 3	26 (38.8)	19 (31.7)		21 (31.8)	
Morphine requirement at PACU	39 (58.2)	27 (45)	0.137	30 (45.5)	0.141
PONV	9 (13.4)	6 (10)	0.550	7 (11)	0.616
Antiemetics requirement at PACU	18 (26.9)	10 (16.7)	0.166	12 (18.2)	0.231

Data are presented as the frequency (per cent). ^1^Chi-square test. NRS, Numerical Rating Scale; PACU, Postanaesthesia Care Unit; PONV, postoperative nausea and vomiting.

## Data Availability

The authors declare that all data used to support the findings of this study were supplied by Faculdade de Medicina da Universiadade do Porto under a license and so cannot be made freely available. Requests for access to these data should be made to the corresponding author.

## Supplementary Materials

Supplementary Materials Supplementary Material S1: Quality of Recovery (QoR)-15 Score. The Quality of Recovery is a survey with 15 items used to evaluate the postoperative period. Supplementary Material S2: Nursing Delirium Screening Scale (Nu-DESC). The Nursing delirium Screening Scale (Nu-DESC) used in the study is a clinical tool to evaluate postoperative emergence delirium (ED). Patients were considered positive for ED with a Nu-DESC ≥2 points.
