# Supplementary material for: Impact of Ketamine on Quality of Recovery after Laparoscopic Surgery: A Single-Centre Single-Blinded Trial Using the QoR-15 Questionnaire
Source: Anesthesiol Res Pract. 2023 Jan 20;2023:8890025. doi: 10.1155/2023/8890025 (PMC9883102; doi:10.1155/2023/8890025)

**Impact of ketamine on quality of recovery after laparoscopic surgery: a single-centre single-blinded trial using the QoR-15 questionnaire.**

*Helder Pereira (HP)^a, b^, Maria Inês Graça (MG)^b^, Diana Fonseca (DF)^b^, Alfredo Mendes-Castro (AMC)^b, c^, Fernando Abelha (FA)^a, b^*

^a^ Department of Surgery and Physiology - Faculty of Medicine, University of Porto, Alameda Professor Hernâni Monteiro, Porto, Portugal (HP, FA).

^b^ Anaesthesia Department, Centro Hospitalar Universitário de São João, Alameda Professor Hernâni Monteiro, Porto, Portugal (HP, MG, DF, AMC, FA).

*^c^* Department of Community Medicine, Information and Health Decision Sciences - Faculty of Medicine, University of Porto, Alameda Professor Hernâni Monteiro, Porto, Portugal.

Corresponding author: Helder Pereira

Faculdade de Medicina da Universidade do Porto, Porto, Portugal

Alameda Professor Hernâni Monteiro, 4200-319 Porto, Portugal

Email: up200904722@med.up.pt

ORCID: *H. Pereira* https://orcid.org/0000-0002-5921-8093

**Supplementary Material**

**S1 – Quality of Recovery (QoR)-15 Score**

The Quality of Recovery (15 item) is a survey used to evaluate the postoperative period.

**S2 – Nursing Delirium Screening Scale (Nu-DESC)**

The Nursing delirium Screening Scale (Nu-DESC) used in the study. Patients were considered positive for emergence delirium with a Nu-DESC > 2 points.


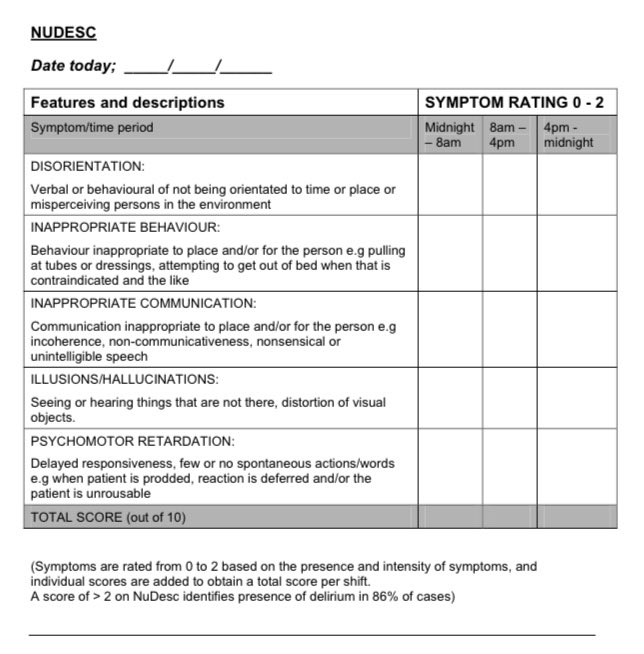

Supplement: Supplementary Materials — Supplementary Material S1: Quality of Recovery (QoR)-15 Score. The Quality of Recovery is a survey with 15 items used to evaluate the postoperative period. Supplementary Material S2: Nursing Delirium Screening Scale (Nu-DESC). The Nursing delirium Screening Scale (Nu-DESC) used in the study is a clinical tool to evaluate postoperative emergence delirium (ED). Patients were considered positive for ED with a Nu-DESC ≥2 points. [file 8890025.f1.docx]
